# Supplementary material for: Phytochemical Study and Antiglioblastoma Activity Assessment of Plectranthus hadiensis (Forssk.) Schweinf. ex Sprenger var. hadiensis Stems
Source: Molecules. 2022 Jun 14;27(12):3813. doi: 10.3390/molecules27123813 (PMC9230782; doi:10.3390/molecules27123813)
Supplement: Supplementary file 1 [file molecules-27-03813-s001.zip › molecules-1763470-supplementary.pdf]

Supplementary Material

1 Supplementary Data (Roy, 1).

1.1. NMR

^{1}H NMR (300 MHz, $CDCl_3$) δ 7.26, 7.21 (1H, s, OH), 5.66 (1H, d, *J* = 2.13 Hz, 7β-H), 4.32 (s, 1H, H-6), 3.17 (1H, sept, *J* = 7,05 Hz, H-15), 2.62 (1H, s, H-6- OH), 2.04 (3H, s,OAc-7α), 1.93 (1H, d, *J* = 3.9 Hz), 1.61 (5H, d, *J* = 5.3 Hz), 1.35 (1H, s), 1.25 (6H, d, *J* = 3.5 Hz), 1.23 (3H, s), 1.21 (3H, d, *J* = 2.7 Hz), 1.19 (2H, s), 0.95 (3H, s), 0.07 (1H, s).

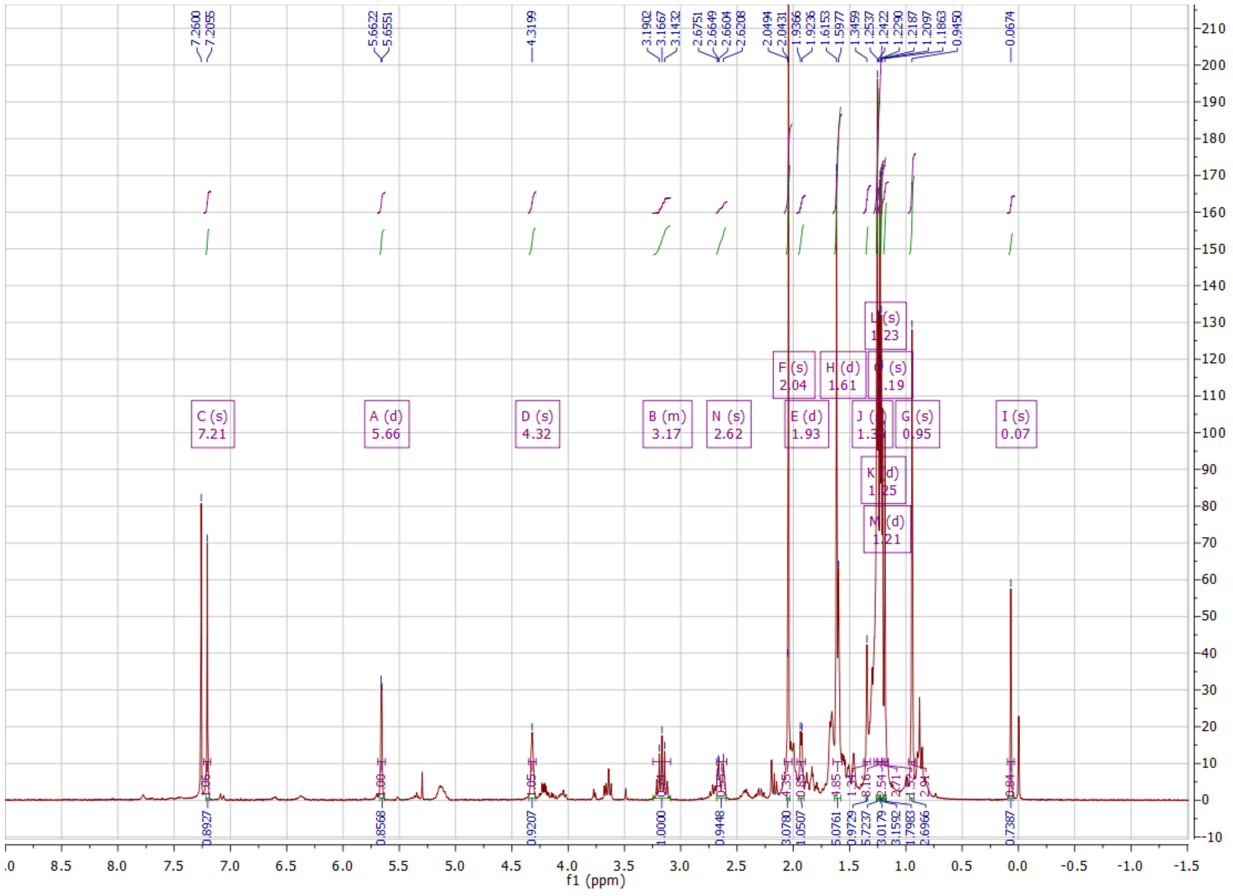

Figure S1: ^{1}H-NMR spectrum of Roy.

1.2. FTIR

IR (cm^{-1}) (representative peaks): 3558.65 (-OH), 1731.58 (C=O), 1236.13 (-O-C-).

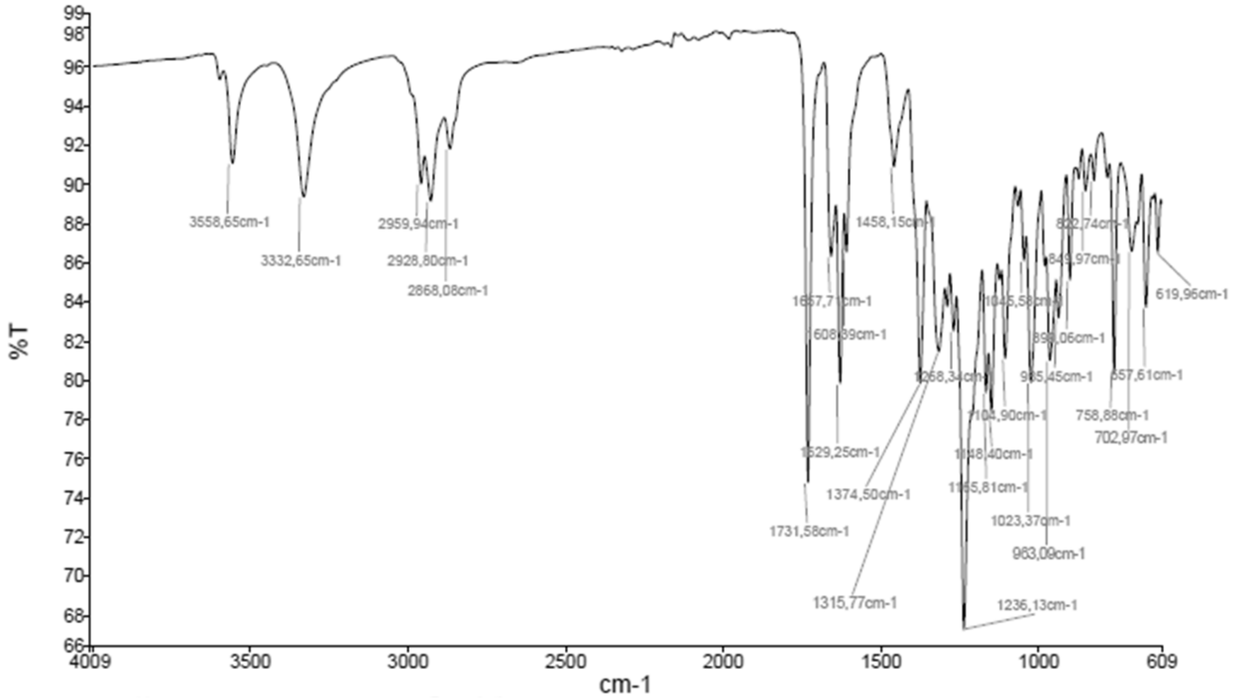

Figure S2: FT-IR spectrum of Roy.

1.3 Calibration curve

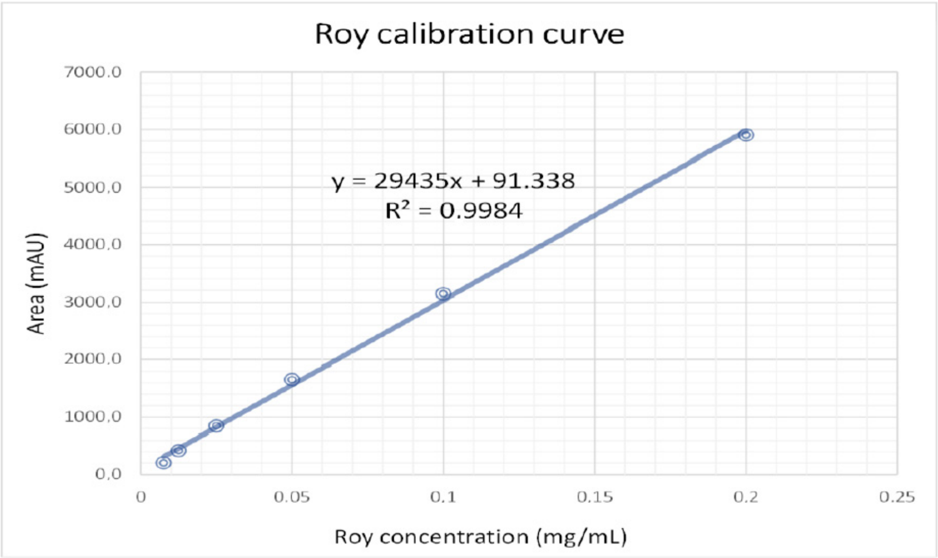

Figure S3: Calibration curve of Roy.

Tables S1: Calibration curve of Roy related tables.

Roy Concentration(mg/mL)	X Area (mAU)	X tr (min)
0.2	5905.0	14.377
0.1	3143.8	18.183
0.05	1654.3	17.604
0.025	846.8	15.273
0.0125	417.9	15.038
0.0075	206.9	14.599

	**0.2 mg/mL**	
	tr (min)	Area (mAU)
	14.513	5853.2
	14.438	5937.2
	14.181	5924.6
∑=	14.377	5905.0

	**0.1 mg/mL**	
	tr(min)	Area (mAU)
	18.841	3119.3
	18.036	3153.1
	17.673	3159.1
∑=	18.183	3143.8

	**0.05 mg/mL**	
	tr(min)	Area (mAU)
	17.646	1575.8
	17.569	1787.5
	17.596	1599.6
∑=	17.604	1654.3

	**0.025 mg/mL**	
	tr(min)	Area (mAU)
	15.262	839.8
	15.386	852.1
	15.171	848.4
∑=	15.273	846.8

	0.0125 mg/mL	
	tr(min)	Area (mAU)
	14.994	414.8
	15.142	418.1
	14.977	420.7
∑=	15.038	417.9

	0.0075 mg/mL	
	tr(min)	Area (mAU)
	14.877	207.4
	14.602	206.0
	14.319	207.3
∑=	14.599	206.9

2 Supplementary Data (DiRoy, 3).

2.1 NMR

Structural Characterization:

^{1}H NMR (300 MHz, $CDCl_3$) δ 7.28 (1H, s, 12-OH), 4.52 (1H,d, J=1.95, H-6 or H-7), 4.46 (1H, s, H-6 or H-7), 3.17 (1H, sept, J = 7,05 Hz, H-15), 2.60 (1H, d, J= 13.29, OH of C6 or C7), 2.18 (1H, m, J= 7.26,), 1.87 (1H, m, J = 3.12 Hz,), 1.82 (1H, dd, J = 2.82 Hz,), 1.61 (5H, d, J=0.69,), 1.43 (6H, m, J = 1.77 Hz,), 1.24 (6H, d, J=5.25,), 1.21 (6H, d, J = 0.84 Hz,), 1.11 (1H,s,), 1.05 (6H,s,), 0.90 (3H, s), 0.88 (6H,s,), 0.85 (7H, m, J=1.47,), 0.83 (3H,s).

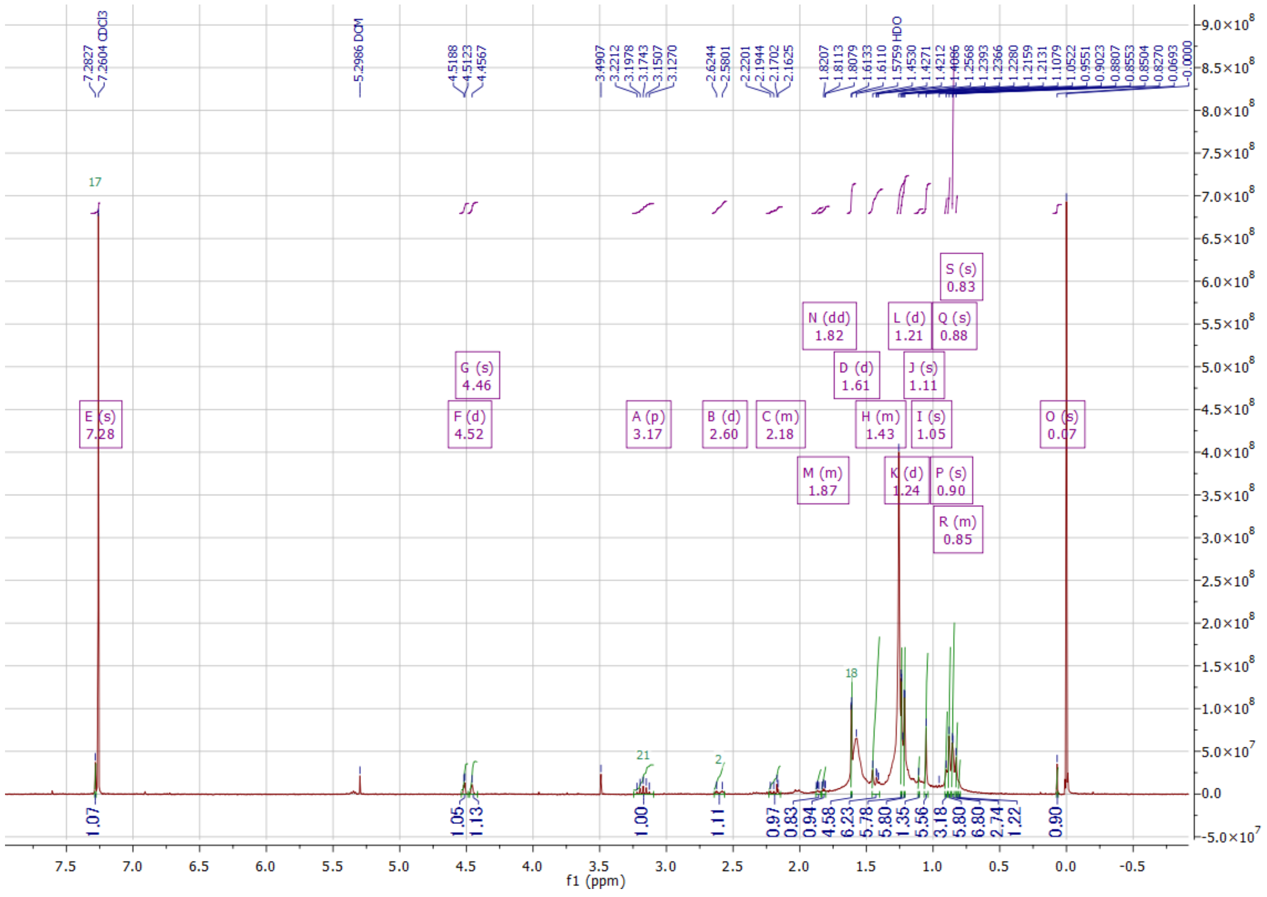

Figure S4: ^{1}H-NMR spectrum of DiRoy.

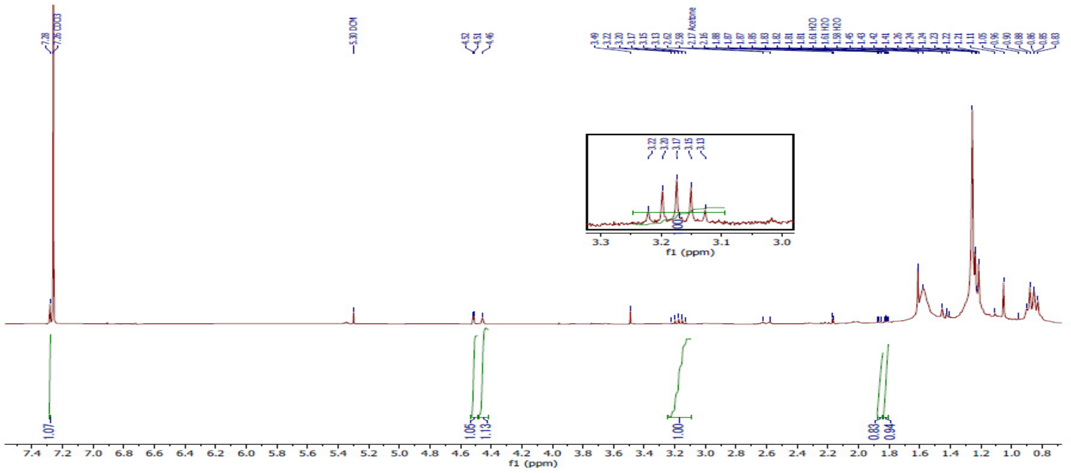

2.2 FTIR

IR (cm^{-1}) (representative peaks): 3438.39, 3337.49 (OH), 1652.89, 1626.05 (C=O), 1252.56 (-O-C-).

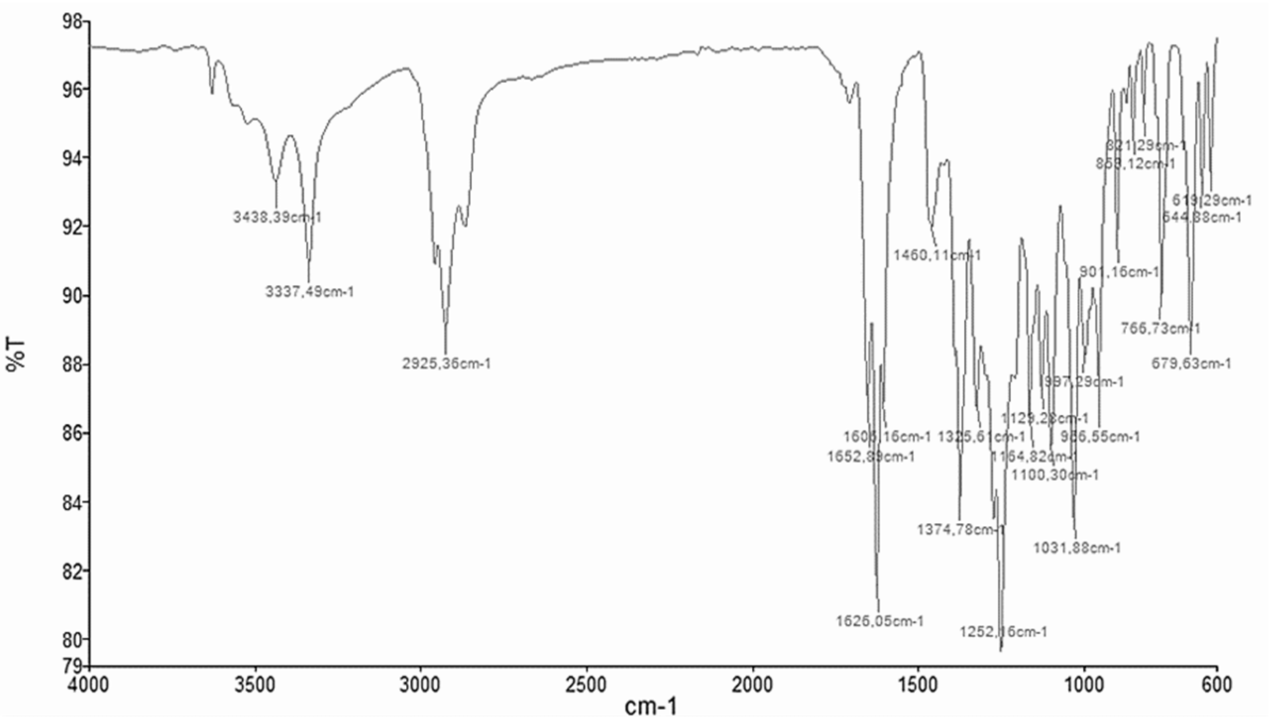

Figure S5: FT-IR spectrum of DiRoy.

2.3 Calibration curve

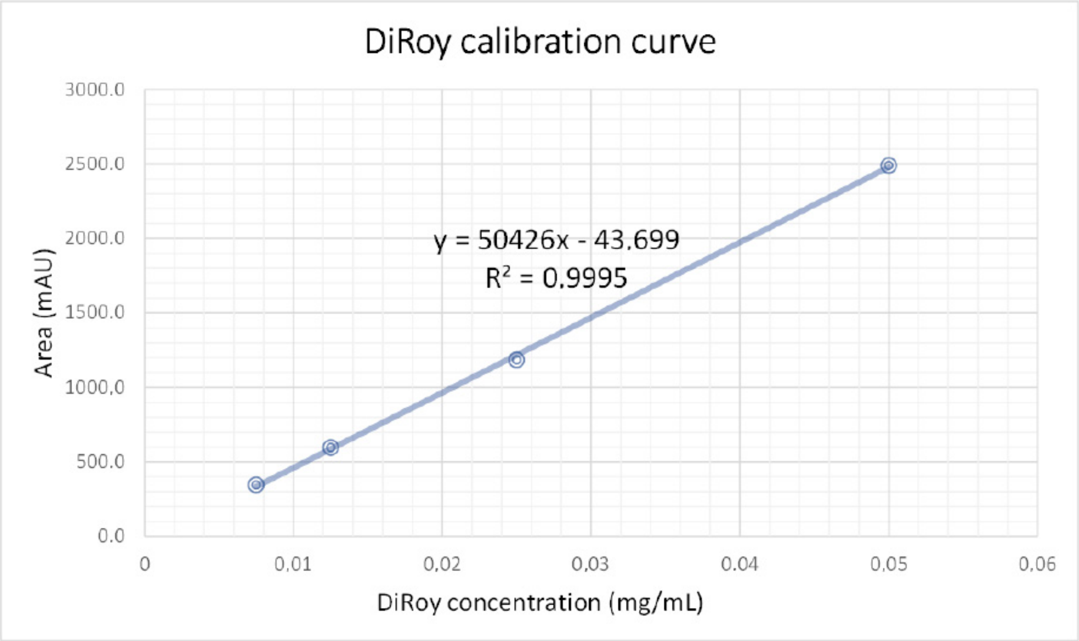

Figure S6: Calibration curve of DiRoy.

Tables S2: Calibration curve of DiRoy related tables.

Injected concentrations of Diroy (mg/mL)	X Area (mAU)	X̄ tr (min)
0.05	2490.1	16.843
0.025	1183.8	15.759
0.0125	596.5	14.830
0.0075	345.2	14.296

	**0.05 mg/mL**	
	tr(min)	Area (mAU)
	17.098	2490.8
	16.537	2492.6
	16.893	2486.8
∑=	16.843	2490.1

	**0.025 mg/mL**	
	tr(min)	Area (mAU)
	16.380	1189.3
	15.710	1191.0
	15.186	1171.1
∑=	15.759	1183.8

	0.0125mg/mL	
	tr(min)	Area (mAU)
	15.154	635.6
	14.669	610.0
	14.668	544.0
∑=	14.830	596.5

	0.0075 mg/mL	
	tr(min)	Area (mAU)
	14.497	388.0
	14.435	330.6
	13.957	317.1
∑=	14.296	345.2

3 HPLC profile fraction V

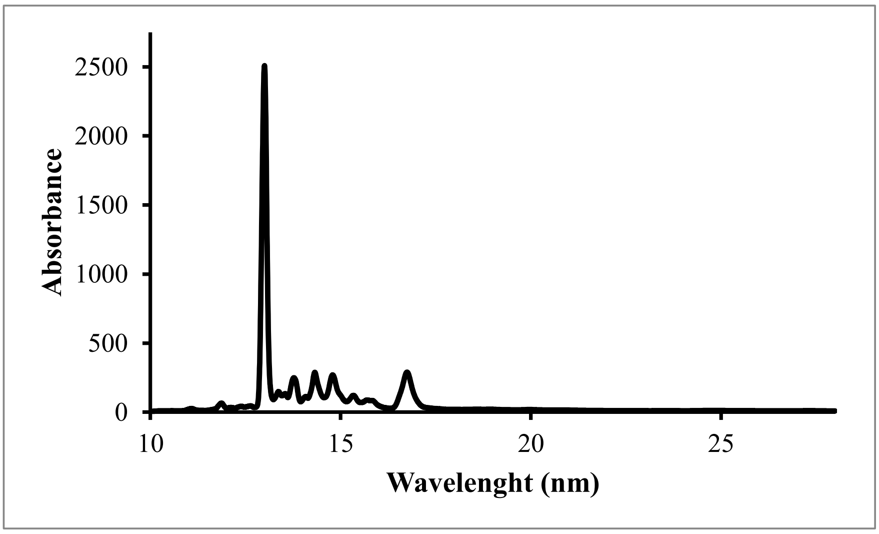

Figure S7. HPLC profile fraction V.

4 UV spectra main compounds

A) Roy (peaks 271, 410 nm)

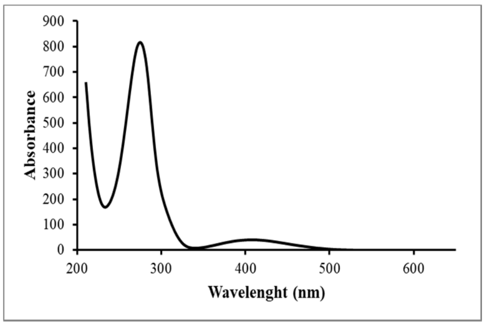

B) DiRoy (peaks 280, 420 nm)

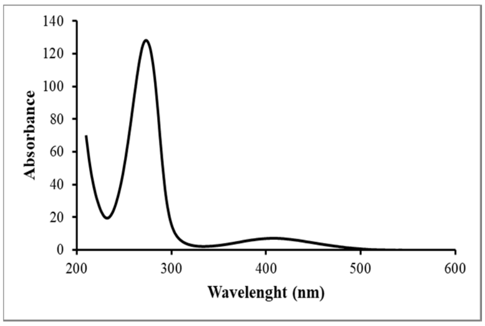

C) Peak B (240,280, 420 nm)

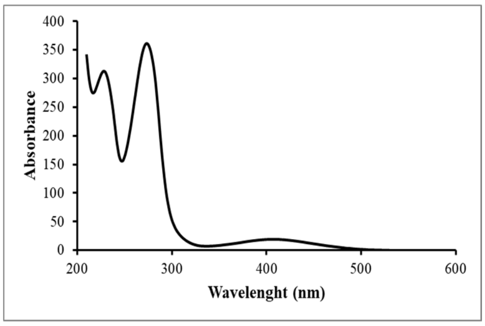

D) Peak C (250,280,350 nm)

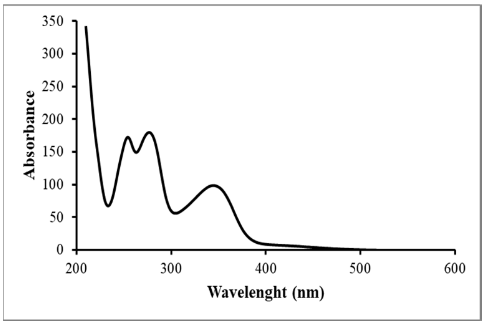

Figure S8. UV spectra of (**A**) 7α-acetoxy-6β-hydroxyroyleanone (Roy,**1**); (**B**) 6β,7β-dihydroxyroyleanone (DiRoy, **3**); (**C**); Peak B, *P. hadiensis* var. hadiensis sems acetone extract. (**D**) Peak C, *P. hadiensis* var. hadiensis sems acetone extract.

5 Supplementary Data BODIPY-7α-acetoxy-6β-hydroxyroyleanone derivative (12)

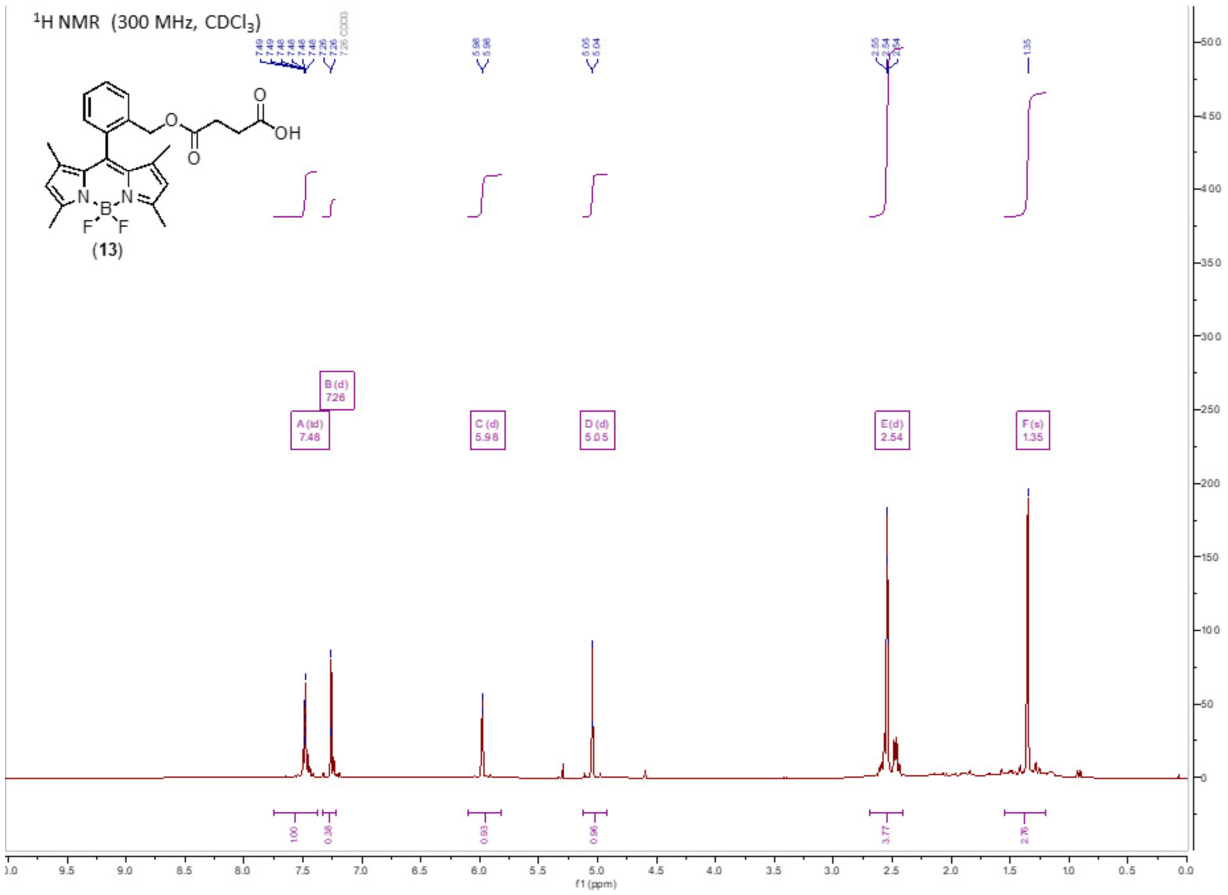

Figure S9. ^{1}H-NMR spectrum (300 MHz) of **(12).**

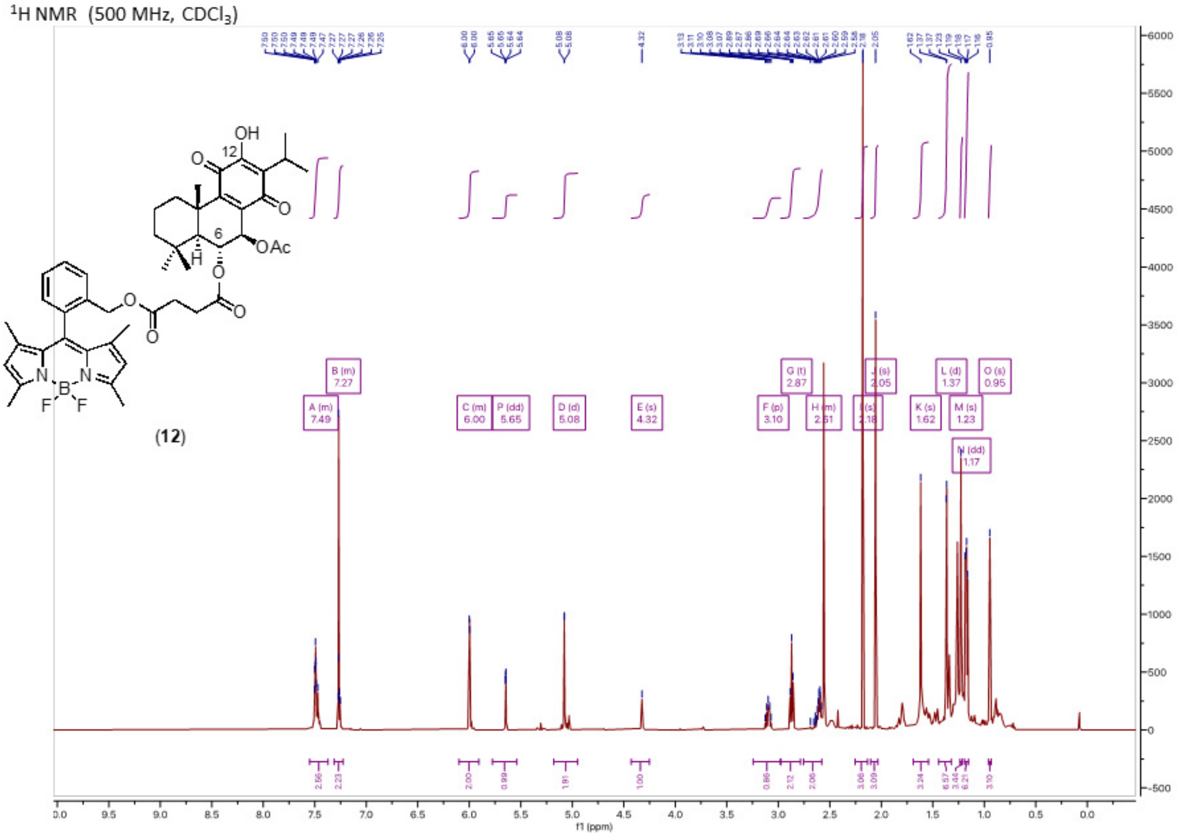

Figure S10. ¹H-NMR spectrum (500 MHz) of **(12)**.

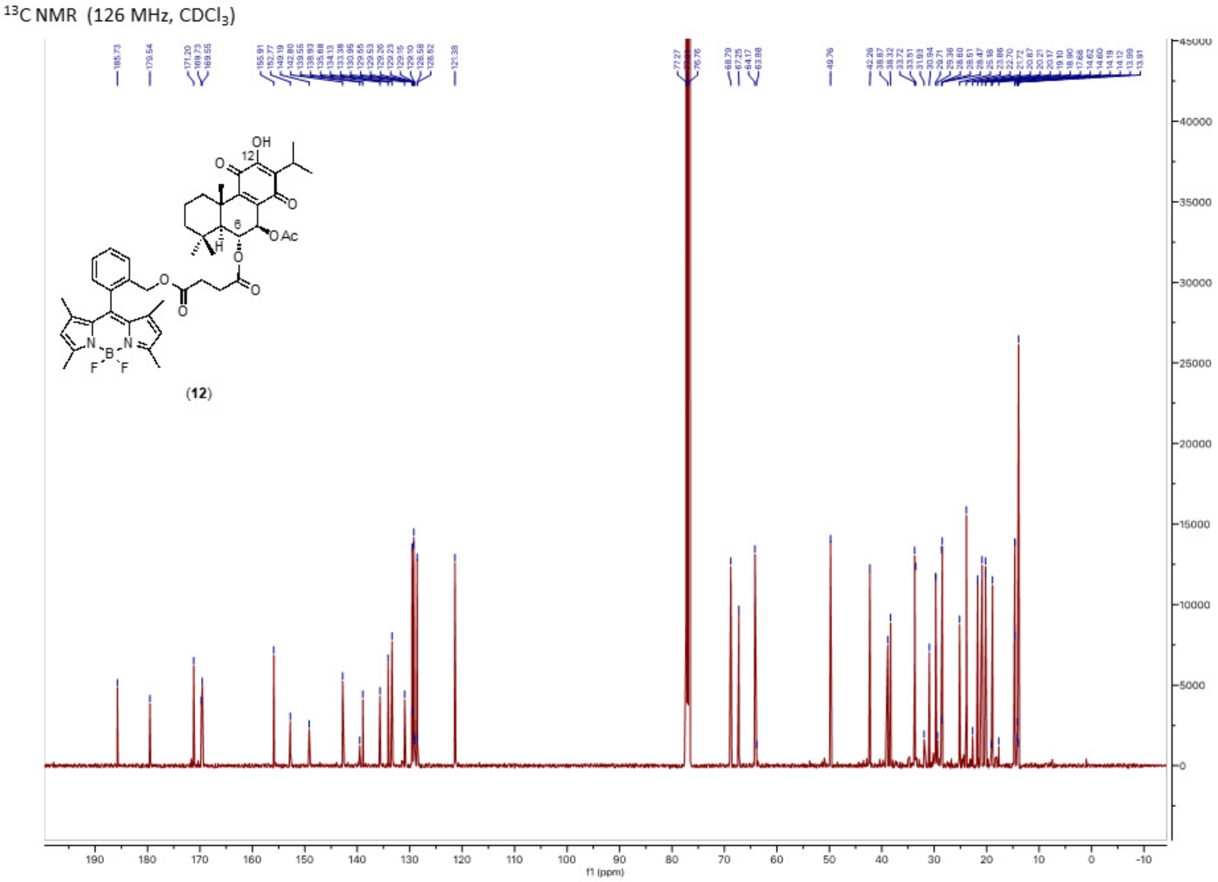

Figure S11. ^{13}C-NMR spectrum (126 MHz) of **(12)**.

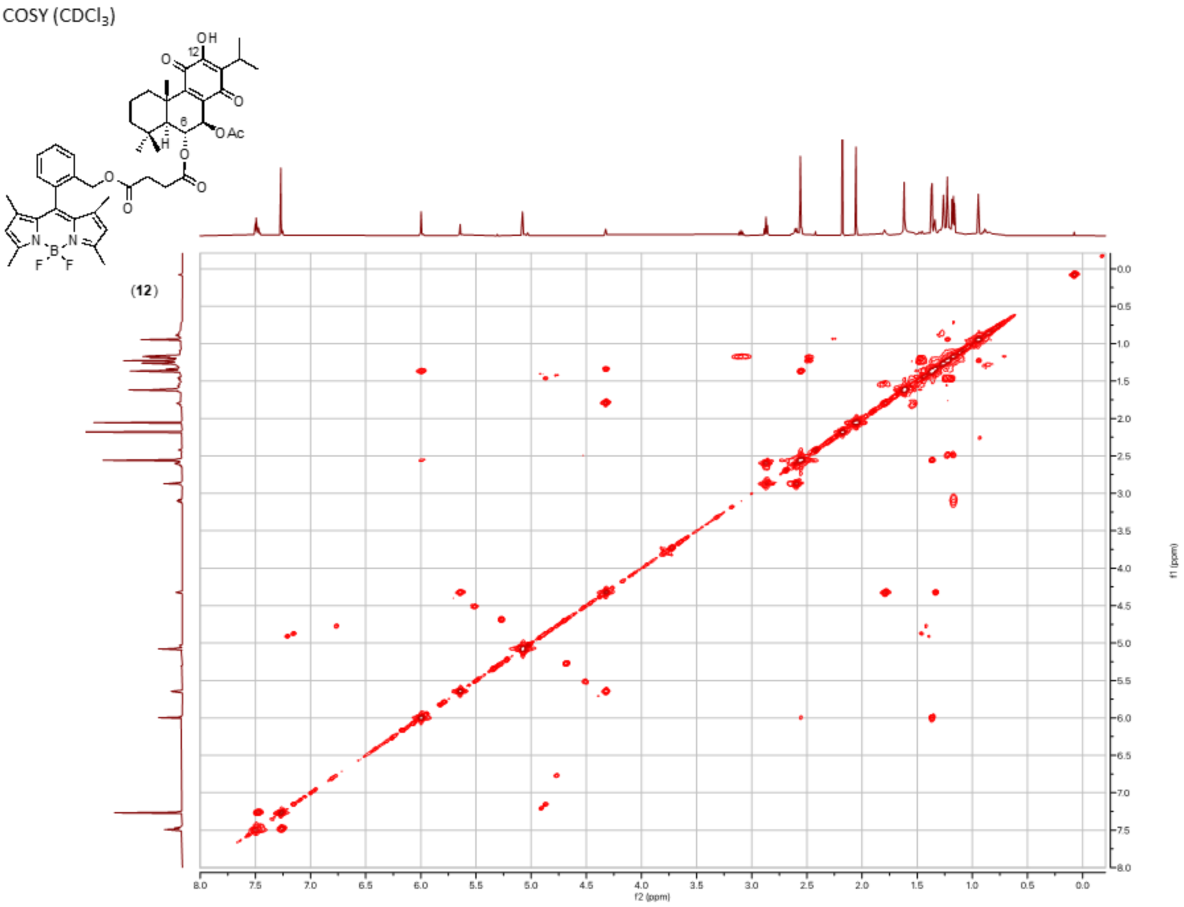

Figure S12. COSY spectrum of **(12)**.

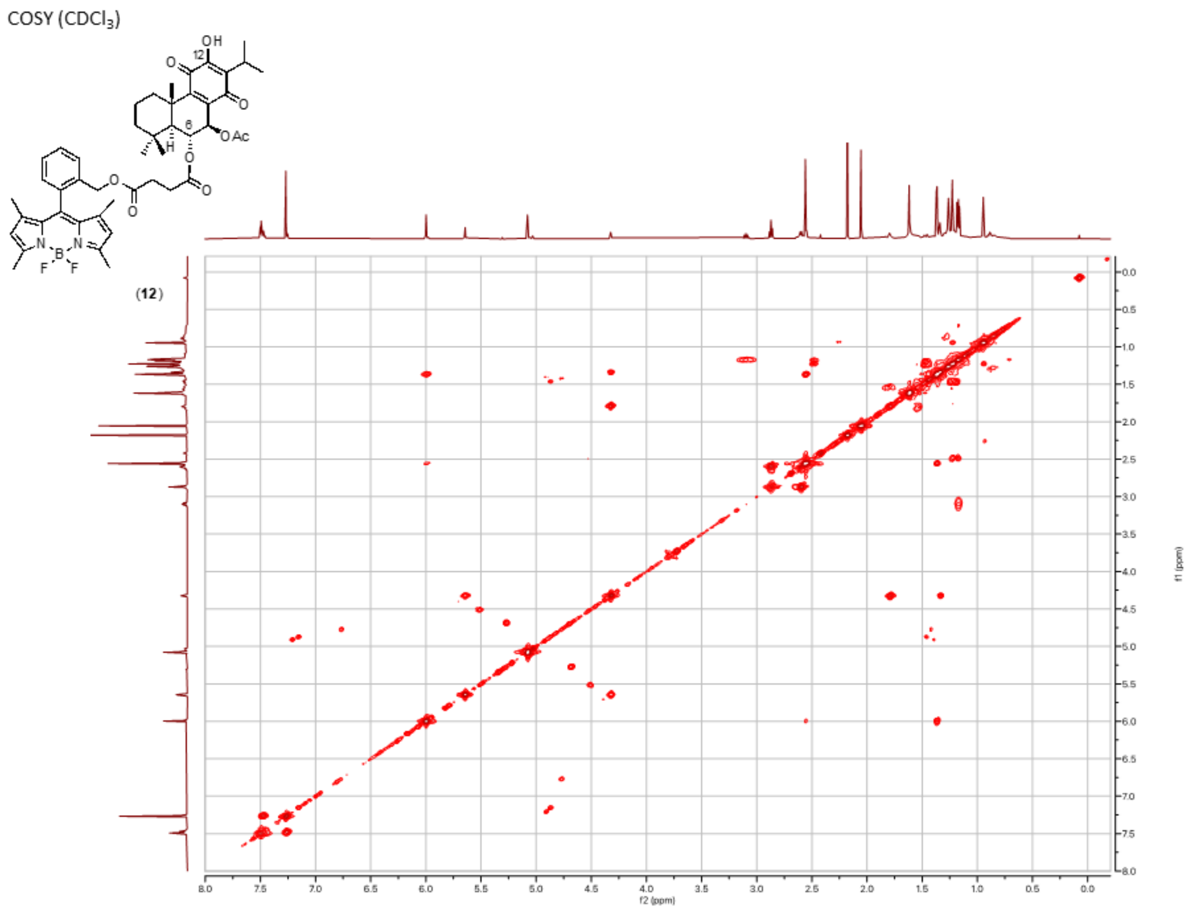

Figure S13. HSQC spectrum of **(12)**.

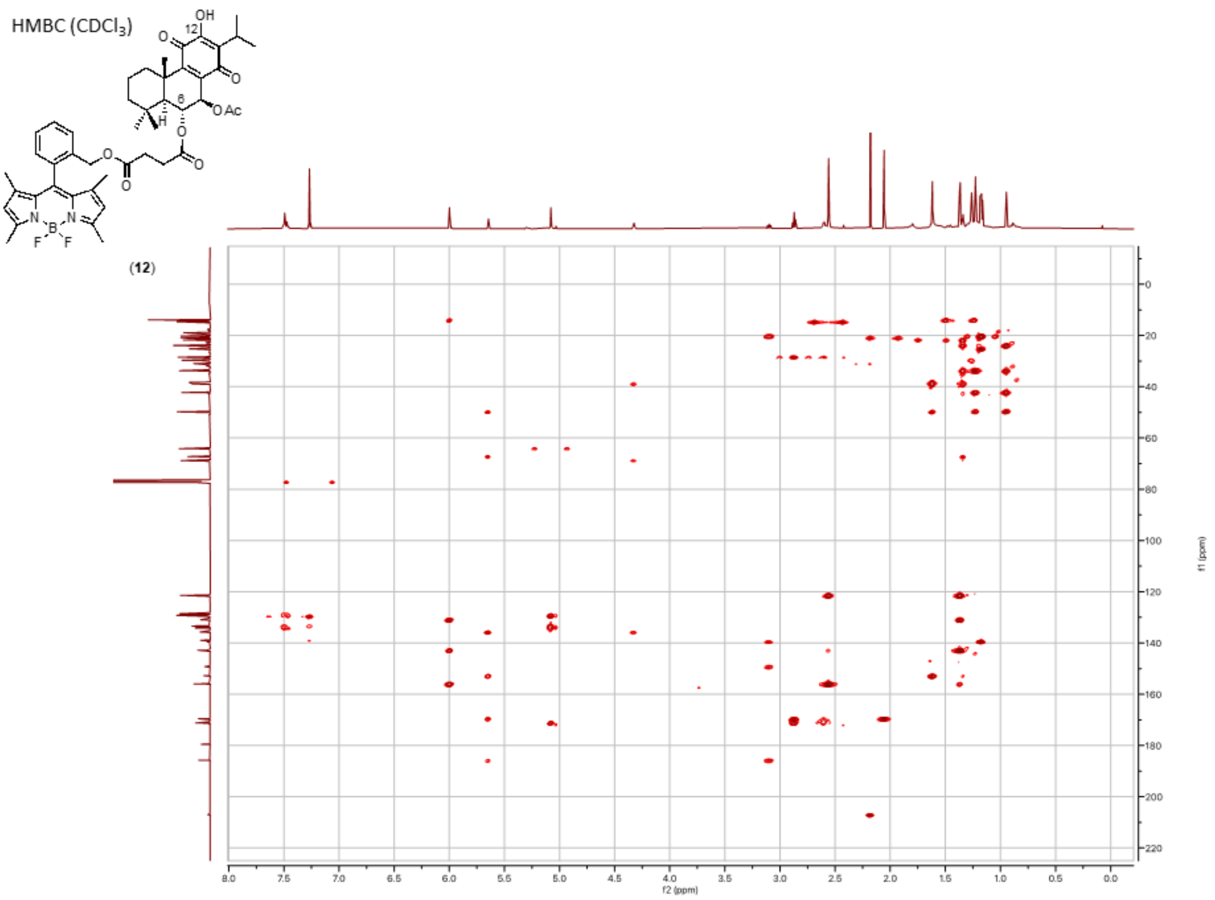

Figure S14. HMBC spectrum of **(12)**.

6. Supplementary Data Cytotoxic effect assays

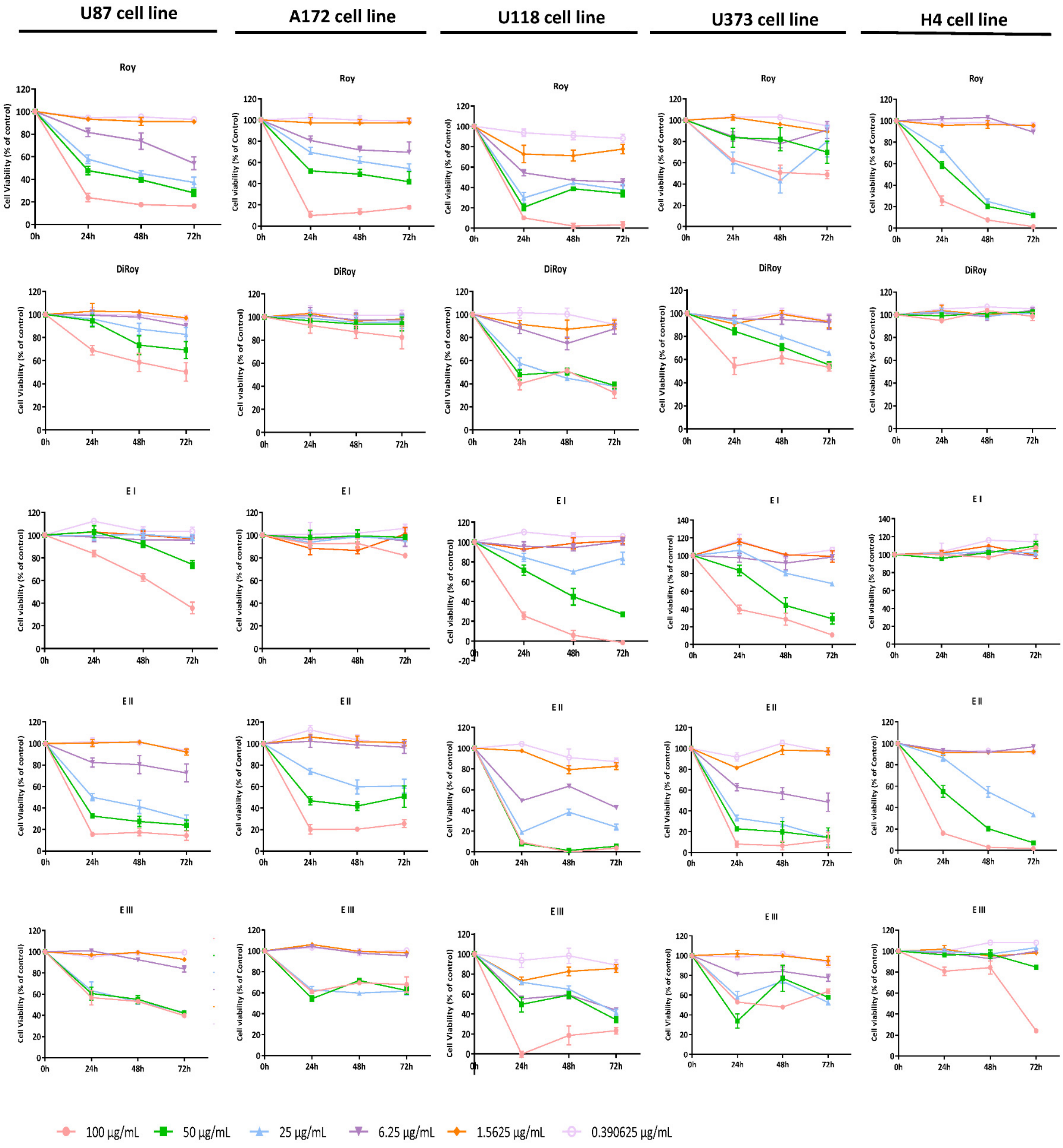
U87 cell line
A172 cell line
U118 cell line
U373 cell line
H4 cell line
Roy
DiRoy
E I
E II
E III
Cell Viability (% of control)
0h
24h
48h
72h
100 µg/mL
50 µg/mL
25 µg/mL
6.25 µg/mL
1.5625 µg/mL
0.390625 µg/mL

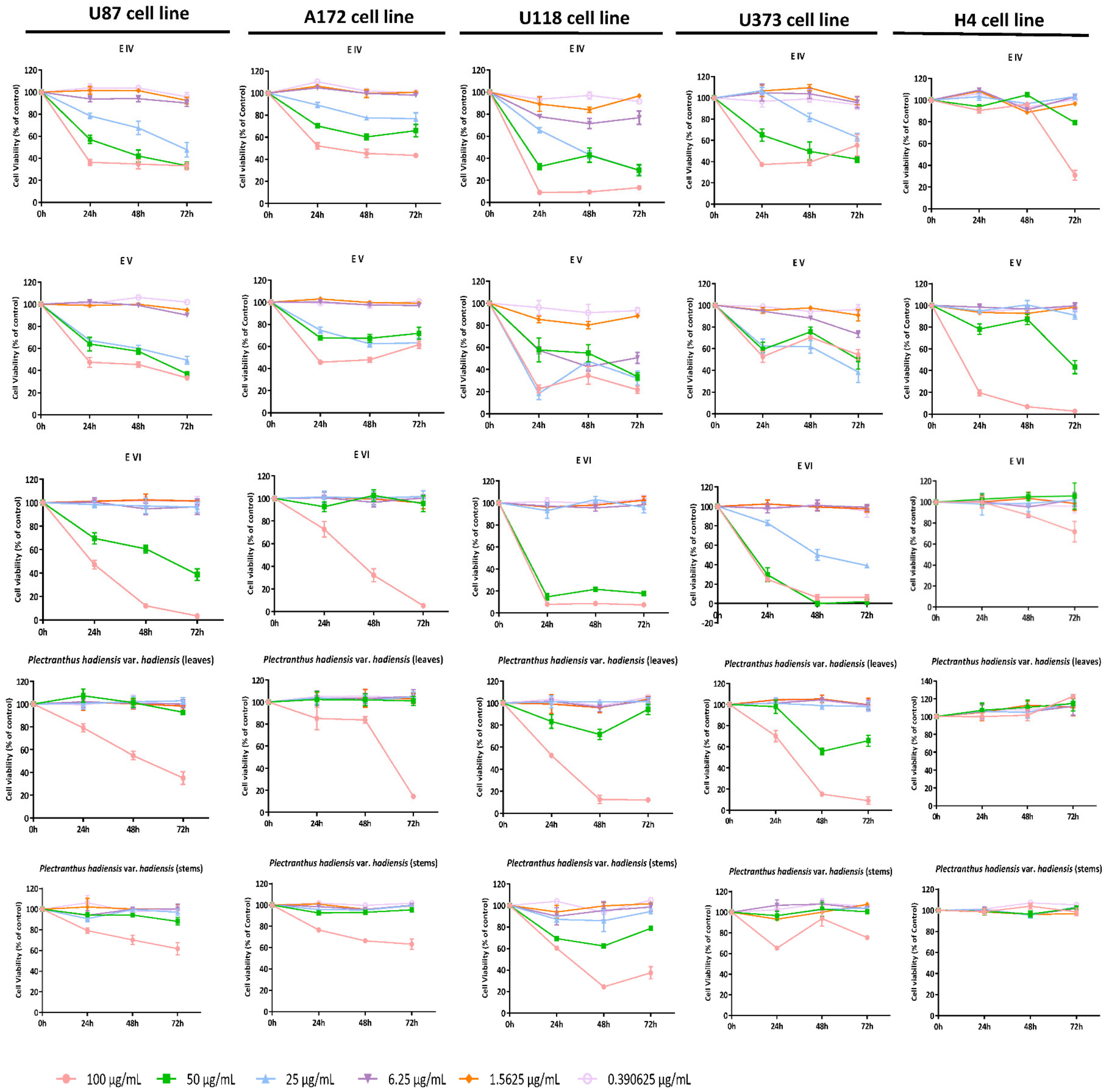

Figure S15. Cytotoxic effect of *Plectranthus hadiensis* var. *hadiensis* phytoproducts, *i.e.*, extracts from stems and leaves, fractions (I, II, III, IV, V and VI), and the isolated compounds (Roy (1) and DiRoy (3)) against U87, A172, U118, U373, and H4 brain tumor cells. Cell cultures were treated with the same compound concentrations for 24, 48 and 72 h and subsequently subjected to the Alamar Blue assay. The cell viability is represented by three independent experiments and is expressed as the percentage of survival compared to untreated control.
